# Effector *Pt*31812 from *Puccinia triticina* acts as avirulence factor for *Lr42*-mediated resistance in wheat

**DOI:** 10.3389/fmicb.2025.1570072

**Published:** 2025-07-18

**Authors:** Jianyuan Li, Jiali Li, Jie Wei, Lin Li, Yue Qi, Yue Zhang, Wenxiang Yang, Qian-Hua Shen

**Affiliations:** ^1^Department of Plant Pathology, College of Plant Protection, Agricultural University of Hebei, Biological Control Center for Plant Diseases and Plant Pests of Hebei, Baoding, China; ^2^College of Biological Sciences and Engineering, Xingtai University, Xingtai, China; ^3^Key Lab of Seed Innovation, Institute of Genetics and Developmental Biology, Chinese Academy of Sciences, Beijing, China; ^4^Department of Agriculture and Animal Husbandry Engineering, Cangzhou Technical College, Cangzhou, China; ^5^Key Laboratory of Plant Resources and China National Botanical Garden, Institute of Botany, Chinese Academy of Sciences, Beijing, China; ^6^Dryland Farming Institute, Hebei Academy of Agricultural and Forestry Science, Hengshui, China

**Keywords:** wheat leaf rust, effector, *Pt*31812, avirulence gene, pathogenesis, *Lr42*

## Abstract

As an obligate biotrophic fungus, the leaf rust pathogen *Puccinia triticina (Pt)* secretes a repertoire of effector proteins into host cells for modulating plant immunity and promoting fungal pathogenesis. Here, we identify the Pt31812 effector and characterize its function in pathogenesis and immune-related activity in plants. In the study, *Pt31812* was cloned by PCR, and the expression pattern and structure were analyzed by qRT-PCR and online softwares. Subcellular localization of Pt31812 was analyzed using transient expression on *Nicotiana Benthamiana*. Further functional analysis was conducted using transient expression and host-induced gene silencing (HIGS). The results showed that *Pt31812* encodes candidate effector with a predicted signaling peptide (SP) at the N-terminus, and its expression was highly up-regulated during *Pt* infection of wheat. Subcellular localization analysis revealed that Pt31812 is localized in cytoplasm and nucleus when expressed in *N. Benthamiana*. Co-expression of Pt31812 and mammalian BAX protein revealed that Pt31812 inhibited BAX-induced cell death in *N. Benthamiana*, and the fragment of 22–88 aa from the N-terminus of the effector was important for the inhibiting activity. Interestingly, expression of Pt31812 in a panel of wheat differential lines with different *Lr* resistance genes showed that Pt31812 specifically triggered cell death in a *Lr42*-harboring wheat line. Furthermore, transient gene silencing of *Pt31812* through BSMV-HIGS approach rendered loss of *Lr42*-mediated resistance against rust race *Pt*.-THSN and altered the infection type from resistant to susceptible. Our data reveal that Pt31812, as a candidate effector with immune inhibiting activity, acts as an avirulence determinant factor during *Pt* infection of *Lr42*-harboring wheat line. These findings highlight immune-related activity of specific *Pt* effectors and lay the foundation for further investigation into mechanisms of leaf rust fungal pathogenesis and recognition.

## 1 Introduction

Plant pathogens secrete a repertoire of effector proteins into host plants to modulate plant immune responses, enabling successful infection and multiplication in plants. Some effectors can either inhibit host immunity and/or sometimes trigger defense responses when recognized by an immune receptors (Zhou and Chai, 2008; Rafiqi et al., 2009). Given their critical roles of effectors in pathogen virulence and in some cases host resistance, the study of pathogen secreted effectors has become a major research focus in the field of plant-pathogen interactions for decades (Lovelace et al., 2023).

Wheat leaf rust, caused by *Puccinia triticina* (*Pt*), is a prominent global wheat disease that seriously threatens food security. Yield losses can reach up to 15–40% or even 70% in a suitable environment (Kolmer, 2005; Huerta-Espino et al., 2011; Savary et al., 2019; Abebe, 2021). *Pt* is a basidiomycete and obligate pathogen with a complex life cycle (Kolmer et al., 2009), encoding hundreds of candidate effector proteins that play key roles in pathogenesis. Many years of studies have identified numerous effectors from rust fungal pathogens, including *Uromyces* sp., (Kemen et al., 2005; Kemen et al., 2013; Pretsch et al., 2013), *Melampsora lini* (Catanzariti et al., 2006; Dodds et al., 2006; Upadhyaya et al., 2014; Anderson et al., 2016), and *Puccinia graminis* sp. *tritici* (*Pgt*) (Salcedo et al., 2017; Chen et al., 2017; Upadhyaya et al., 2021; Outram et al., 2024), as well as from *Puccinia striiformis* sp. *tritici* (*Pst*) PS87 (Gu et al., 2011; Tang, 2013; Dagvadorj et al., 2017; Cheng et al., 2017; Liu et al., 2016; Wang et al., 2016), and so on. Previously, some candidate avirulence effector genes have also been identified from *Pt*, such as *Pt*27 and *Pt*3 (Segovia et al., 2016), and potential avirulence effector genes corresponding to *Lr*20 (Wu et al., 2017). Several *M. lini* effectors, such as AvrM and AvrL567, are secreted from haustorium and specifically recognized by matching plant R proteins to activate defense response (Catanzariti et al., 2006). Some *Pgt* effectors such as PGTAUSPE-10-1, AvrSr27, AvrSr35, and AvrSr50 were identified as the avirulence factors corresponding to resistance protein Sr22, Sr27, Sr35, and Sr50, respectively (Upadhyaya et al., 2014; Upadhyaya et al., 2021; Outram et al., 2024; Salcedo et al., 2017; Chen et al., 2017). Heterologous expression system has been used to determine the subcellular localization of many of these effectors (Lorrain et al., 2018). For example, *Pst* effector proteins Pec6 and PNPi has been shown to localize in the nucleus and cytoplasm in plant cells, with their host targets being identified and co-localized (Liu et al., 2016; Wang et al., 2016).

However, compared with *M. lini*, *Uromyces* sp., and other wheat rust fungi, research on the effector proteins of wheat leaf rust has remained in its early stages. There are few reports on the molecular mechanisms underlying the regulation of wheat immunity by *Pt* effector proteins. For instance, Pt_21 inhibits wheat resistance against leaf rust by interacting with TaTLP1 (Wang et al., 2023). Moreover, some *Pt* effectors have been found to act as avirulent effectors in wheat lines containing matching *Lr* genes, such as Pt13024, AvrLr15 and Pt1641 corresponding to TcLr30, TcLr15 and TcLr1, respectively (Qi et al., 2023; Cui et al., 2024; Chang et al., 2024; Wang et al., 2024). Furthermore, Pt1234 modulates wheat immunity by interaction with the TaNAC069 transcription factor through its C subdomain (Geng et al., 2024). Extensive genome and transcriptome data and effective effector identification methods is crucial to accelerate research and advance our understanding of the pathogenic mechanisms of wheat leaf rust.

Previously, a transcriptome library of wheat leaf rust isolates 13-5-72-1(THSN) was constructed, and 635 candidate effectors from different races of *Pt* were identified through bioinformatics screening by the wheat leaf rust research group at Hebei Agricultural University (HEBAU) (Zhang et al., 2020). Among the 635 candidate effectors, *Pt*31812 was highly expressed during the haustorial formation stages. We investigated the function of Pt31812 in promoting virulence and immune-related activity in plants. The avirulence activity was examined by expressing *Pt*31812 in leaves of a panel of wheat differential lines, using BSMV-HIGS approach to further confirm its role as an avirulence determinant factor in a *Lr42*-harboring wheat line. Our findings lay the foundation for further investigation into mechanisms of leaf rust fungal pathogenesis and pathogen recognition by wheat.

## 2 Materials and methods

### 2.1 Plant materials and strains

Wheat differential lines harboring 41 different genes in the Thatcher background, respectively, including TcLr1, TcLr2a, TcLr2c, TcLr3, TcLr9, TcLr16, TcLr24, TcLr26, TcLr3ka, TcLr11, TcLr17, TcLr30, TcLrB, TcLr10, TcLr14a, TcLr18, TcLr21, TcLr28, Lr42, TcLr2b, TcLr3bg, TcLr14b, TcLr15, TcLr19, TcLr20, TcLr23, TcLr25, TcLr29, TcLr27 + 31, TcLr32, TcLr33, TcLr33 + 34, TcLr36, TcLr38, TcLr41, TcLr44, TcLr45, TcLr47, TcLr50, TcLr51, and TcLr53, along with the susceptible line Thatcher and *Pt* race 13-5-72-1 (THSN) were preserved in our lab at HEBAU.

*Agrobacterium tumefaciens* strains GV3101 and EHA105, and the recombinant potato X virus vector pGR107 were generously provided by Professor Wenxian Sun of the China Agricultural University. Barley stripe mosaic virus BSMV-VIGS vectors pCaBS-α, pCaBS-β, pCaBS-γbLIC and pCaBS-γbPDS were presented by Professor Dawei Li of China Agricultural University.

### 2.2 Wheat inoculation using leaf rust urediniospores

10–14-day-old wheat seedlings (*Triticum aestivum* cv. ‘Thatcher’, susceptible genotype), was inoculated with fresh urediniospores of *Puccinia triticina* (Pt) race THSN. Inoculation was performed by evenly dusting spores onto the surface of primary leaves, followed by misting with sterile water. Subsequently, plants were grown under controlled conditions with a diurnal cycle of 16 h light (23°C)/8 h darkness (18°C) at 80% relative humidity.

### 2.3 Cloning and plasmid construction

Total RNA was extracted from wheat leaves inoculated with *Pt* race THSN using TaKaRa MiniBEST Plant RNA Extraction Kit, and cDNA was synthesized using a Reverse-Transcription System (Abm, Canada). *Pt31812* sequence was amplified from the cDNA with specific primers (Supplementary Table S1), and subcloned into pGR107 vector through restriction enzyme digestion and ligation for agroinfiltration in *N. benthamiana* or wheat, all candidates confirmed by sequencing.

### 2.4 Bioinformatics analysis

Signal peptides, mitochondrial/chloroplast targeting signals, and transmembrane domains were predicted using SignalP v4.1,^1^ TargetP v1.1,^2^ and TMHMM v2.0 (transmembrane prediction using hidden Markov models),^3^ respectively. The cysteine residue content was analyzed, the conserved domain was indentified using the Pfam database,^4^ and *de novo* motif prediction and analysis of novel sequence motifs using MEME.^5^ Conserved [Y/F/W]xC motifs were detected using Perl softare.

### 2.5 Quantitative real-time PCR (qRT-PCR)

Total RNA was isolated from wheat leaves at 0, 6, 12, 18, 24, 36, 48, 60, 72, 96, and 120 hpi infected at with *Pt* race THSN. To assess the transcript level of the *Pt31812* gene during *Pt* infection, qRT-PCR was conducted using the LightCycler^®^ 96 real-time PCR system (Roche) with indicated primer (Supplementary Table S1), and the elongation factor 1-alpha gene (*EF1a*) serving as the reference gene. Relative gene expression was quantified by comparative 2^–ΔΔ*Ct*^ method, with statistical significance determined by Student’s *t*-test. All experiment were performed with three independent biological replicates.

### 2.6 Confocal laser scanning microscopy and localization analysis

For subcellular localization analysis, the coding sequences of *Pt31812*(△SP) was subcloned into vector pGR107 containing green fluorescent protein (GFP) to genrated pGR107-△ Pt31812-GFP. The pGR107-△ Pt31812-GFP and GFP alone (pGR107-GFP) were transformed into *A. tumefaciens* strain GV3101 and infiltrated into *N. benthamiana* leaves. The confocol imaging was conducted at 48 h post infiltration, with GFP excitation set at 488 nm. (OLYMPUS BX51, Japan).

### 2.7 *Agrobacterium*-mediated transient expression in *N. benthamiana*

The recombinant vectors pGR107-Pt31812, pGR107-Pt31812-ΔSP, a series of Pt31812 variants and BAX were transformed into *A. tumefaciens* strain GV3101. Agrobacteria were cultured overnight at 28°C at 220 rpm in LB medium, then resuspended in 10 mM MgCl_2_ to a final OD_600_ = 0.5, and incubated in dark at room temperature for 2–3 h. Equal volumes of Pt31812 constructs (or its variants) and BAX were mixed and infiltrated into *N. benthamiana* leaves (Yuan et al., 2011). The cell death symptoms were observed and photographed at 5 days post-infiltration.

### 2.8 *Agrobacterium*-mediated transient expression in wheat

The pGR107-Pt31812 construct was transformed into the *A. tumefaciens* strain GV3101, and generated Agrobacterium suspension as previously described in 2.7, and infiltrated into second leaf of wheat. Cell death symptoms were observed at 5–7 days post-infiltration.

### 2.9 Barley stripe mosaic virus-mediated gene silencing in wheat

Barley stripemosaic virus-mediated gene silencing in wheat was modificated with previously described by Cheng et al. (2017). A 261 bp cDNA fragment of *Pt31812* was subcloned into pCaBS-γbLIC vector to generated pCaBS-γbLIC-Pt31812. pCaBS-α, pCaBS-β and pCaBS-γbLIC construct were transformed into *A. tumefaciens* strain EHA105, respectively. The agrobacteria were resuspended in infiltration buffer to OD600 = 1.0 and mixed at 1:1:1 ratio to infiltrate *N.benthamiana*. After 10 days, *N.benthamiana* leaf sap was extracted by grounding in 20 mM Na-phosphate buffer (pH 7.2) containing 1% celite. to inoculated two-leaf stage wheat leaves (Yuan et al., 2011). The newly emerged leaves with viral phenotypes were infected with *Pt* race THSN. The infection types were identificated (Roelfs and Martens, 1988) and recorded at 10 dpi.

### 2.10 Histological observations

Leaf samples collected at 24, 48, and 120 hpi were stained with Fluorescent Brightener 28 (FB 28) following a modified protocol (Kang et al., 2003). Briefly, samples were first fixed in methanol-chloroform (1:2 v/v) for 6 h, boiled in lactophenol oil-95% ethanol (1:2 v/v) for 1.5 min, and incubated overnight. After sequential washing with 50% ethanol for 30 min and ddH2 O, the samples were treated with 0.5 M sodium hydroxide for 30 min followed by ddH_2_O rinses. Subsequently, the samples were soaked in 0.1 M Tris-HCl buffer (pH 8.5) for 30 min before staining with 0.1% FB 28 solution for 5 min. Following several ddH_2_O washes, the stained samples were preserved in 25% glycerol (v/v) for microscopic observation. The hyphal were visualized using a laser scanning confocal microscope (OLYMPUS FV1000, Japan) with excitation at 405 nm and emission and 488 nm (Wang et al., 2014).

## 3 Results

### 3.1 Bioinformatics analysis of Pt31812 sequence

Among the 635 candidate effectors of wheat leaf rust fungus, Pt31812 is one of the candidate effectors whose gene expression were highly induced during haustorial stage (Zhang et al., 2020). To further characterize this effector, we amplified the cDNA sequence of *Pt31812* from RNA samples derived from the *Pt* strain THSN, which encodes a candidate effector of 208 amino acids and harbors a 22-aa N-terminal signal peptide (SP), predicted by SignalP v4.1. Further TargetP v2.0 analysis indicated no mitochondria/chloroplast targeting domain, and TMHMM predicted no transmembrane domain, in the effector sequence. Further searching against PfamA and PfamB libraries and NCBI conserved domain database also revealed no known conserved domains, and MEME Suite also did not identify any new motif in Pt31812. Interesting, a conserved [Y/F/W]xC motif characteristic of powdery mildew effectors was identified in Pt31812 by using Perl software (Table 1).

**TABLE 1:** The best hit of the effector Pt31812 in NCBI database through BlastX analyses.

Gene	Size (aa)	Cys	Best hit in NCBI database	*E*-value	Pfam motif	Domain
*Pt31812*	208	9	hypothetical protein PTTG_06577	2.E-155	NO	YxC

### 3.2 *Pt31812* expression is highly induced during *Pt*. infection of wheat

**FIGURE 1:**
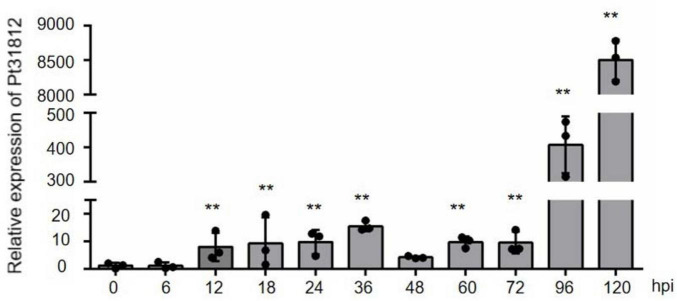
*Pt31812* expression is highly up-regulated during *Pt.* infection of wheat. *Pt31812* transcript level were analyzed by qRT-PCR analysis during *Pt.* infection of leaves of wheat cv. Thatcher from 0 to 120 hpi. Relative expression was calculated by the comparative Ct method with *EF1* as a reference gene. Error bars = ± SD (*n* = 3 for biological replicates). ** for *P* < 0.01; Statistic analysis was performed by Student’s *t*-test.

To analyze the expression pattern of *Pt31812* during infection of wheat by *Pt.-*THSN isolate, a time-course experiment was performed and data analyzed by qRT-PCR analysis. Expression of *Pt31812* was markedly induced at 12 hpi and reached a pick at 36 hpi, and was highly induced later at 96 and 120 hpi (Figure 1).

### 3.3 Pt31812 is localized to the cytoplasm and nucleus in *N. benthamiana*

**FIGURE 2:**
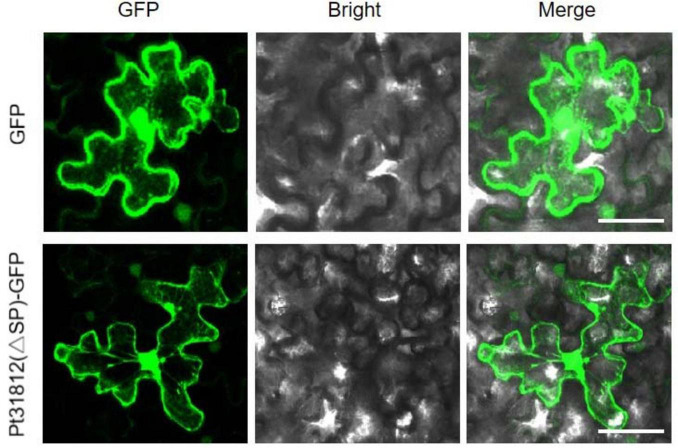
Pt31812 is localized in cytoplasm and nucleus in *N. benthamiana.* GFP and Pt31812(△SP)-GFP fusion proteins were transiently expressed in *N. benthamiana* via *Agrobacterium*-infiltration. Confocal imaging was done at 48 hpi. Scale bar = 5 μm.

To examine the subcellular localization of Pt31812, we generated construct of GFP fusion of Pt31812 (lacking SP). Pt31812(△SP)-GFP and GFP alone were individually expressed in *N. benthamiana* by *Agrobacterium-*infiltration. Confocal imaging showed that Pt31812(△SP)-GFP was distributed in the cytoplasm and nucleus, similar to the control GFP (Figure 2), indicating Pt31812 is localized to both the cytoplasm and nucleus in plant cells.

### 3.4 Pt31812 inhibits BAX-induced cell death in *N. benthamiana*

**FIGURE 3:**
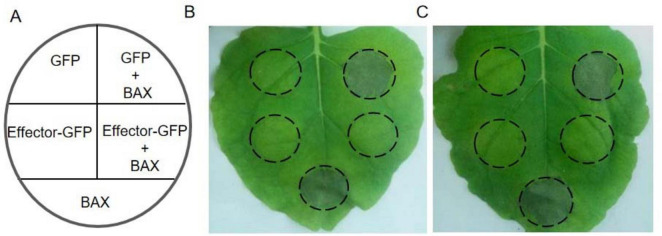
Pt31812 suppresses BAX-induced cell death in *N. benthamiana*. (A) Scheme of *Agrobacterium*-infiltration. (B,C) Expression of GFP or effector-GFP fusion alone (left half), or coexpression with BAX (right half) in leaves of *N. benthamiana*, and Effector-GFP fusion represents Pt31812(FL)-GFP (B) or Pt31812(△SP)-GFP (C). The cell death phenotype was photographed at 5 d post infiltration.

BAX is a pro-apoptotic protein of the Bcl-2 family in mouse that can trigger hypersensitive responsive (HR)-like cell death in plants (Lacomme and Santa, 1999). To examine the activity of Pt31812 in inducing cell-death or inhibiting BAX-induced cell death in plants, we performed *Agrobacterium*-mediated transient gene expression in *N. benthamiana* according to the scheme (Figure 3A). Leaves of *N. benthaminana* expressing Pt31812-GFP alone or co-expressing Pt31812-GFP and BAX were observed at 5-days after infiltration. As shown in Figure 3B, BAX alone induced obvious cell death, whereas co-expression of Pt31812(FL)-GFP and BAX resulted in complete suppression of cell death, and co-expression of GFP and BAX led to strong cell death. Similarly, co-expression of Pt31812(△SP)-GFP and BAX also led to complete suppression of cell death (Figure 3C). These results suggest that Pt31812 can effectively inhibit BAX-induced cell death in plants.

### 3.5 A portion of Pt31812 is important for cell death suppression in plants

**FIGURE 4:**
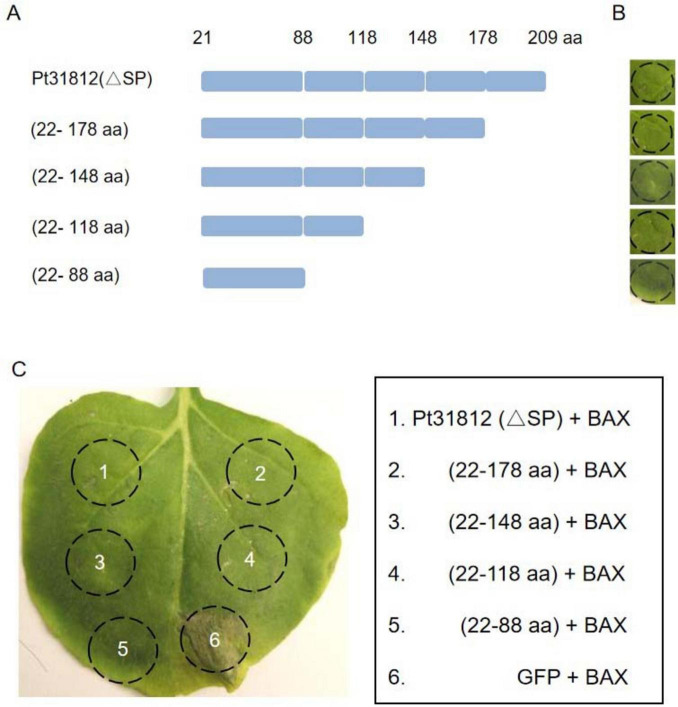
The region of 22–88 aa in Pt31812 is important for suppressing BAX-induced cell death. (A) Schematic representation of Pt31812 deletion variants. (B) expression of Pt31812 deletion variants did not induce cell death upon transient expression in *N. benthamiana* via *Agrobacterium*-infiltration. (C) Co-expression of Pt31812 deletion variants with BAX in *N. benthamiana* leaves, with GFP as a control. Images were taken at 5 dpi.

To further identify the region required for cell-death suppression in Pt31812, a series of deletion mutants of the effector was constructed (Figure 4A). Transient expression of full-length or deletion variants of Pt31812 indicated that Pt31812-FL or Pt31812 variants does not induce cell death in *N. benthamiana* (Figures 4A,B). However, co-expression of full-length or deletion variants of Pt31812 with BAX in *N. benthaminana* resulted cell-death suppression, as compared with co-expression of GFP with BAX (Figure 4C). The shortest fragment we tested is Pt31812 (22–88 aa) that could consistently suppress BAX-induced cell death. These results suggest that the activity of Pt31812 in suppressing BAX-induced cell death relies on the 22–88 aa region, and the SP is not essential for Pt31812 cell-death suppression in plant cells.

### 3.6 Pt31812 induces cell death in *Lr42*-containing wheat line

**FIGURE 5:**
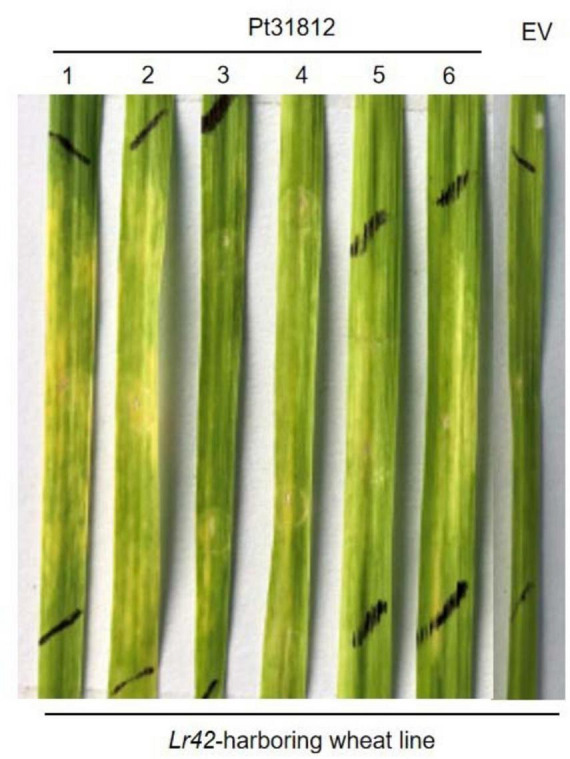
Expression of Pt31812 induces cell death in a *Lr42*-harboring wheat line. Transient expression of Pt31812 or empty vector (EV) in leave of *Lr42*-harboring wheat via *Agrobacterium* infiltration. Representative images were photographed 7 dpi. The two black lines mark the *Agrobacterium*-infiltrated region in the leaf.

To investigate the role of Pt31812 in relation to wheat immunity and/or rust pathogen virulence, we expressed Pt31812 in a panel of wheat differential lines via *Agrobacterium*-infiltration, each of the wheat lines harboring at least a differential leaf rust resistant gene (Qi et al., 2023). Interestingly, among the 41 differential lines we tested, expression of *Pt31812* triggered apparent cell-death phenotype in leaves of the *Lr42*-harboring wheat line (Figure 5; Supplementary Figure S2). Moreover, this Pt31812-induced cell-death phenotype was reproducible in leaves of the *Lr42*-harboring wheat line, which was not observed for the empty vector (EV), at 7 days post infiltration (Figure 5). These results suggest that Pt31812 may specifically induce cell death in *Lr42*-harboring wheat line.

### 3.7 Pt31812 acts as an avirulence factor during *Pt.*-THSN infection of *Lr42*-wheat line

**FIGURE 6:**
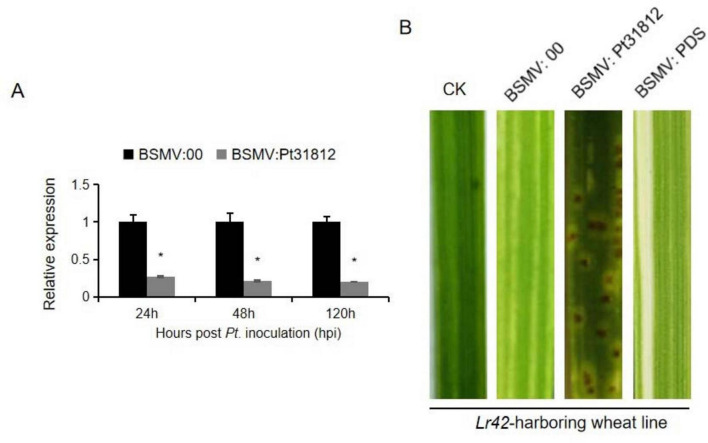
Silencing of *Pt31812* converts avirulent *Pt.*-THSN to a virulent isolate in *Lr42*-harboring wheat line. (A) Transcript levels of *Pt31812* were determined by qRT-PCR at 24, 48, and 120 hpi of *Pt.* isolate in BSMV:00 or BSMV:Pt31812 treated leaves of a *Lr42*-harboring wheat line. * for *P* < 0.05. (B) Disease phenotypes of *Pt.*-THSN infected leaves upon BSMV treatment. The second leaves of *Lr42*-harboring wheat line were treated by sodium phosphate buffer (CK), or BSMV-HIGS vector (BSMV:Pt31812), or empty vector control (BSMV:00). Images were taken at 10 dpi.

To further understand the nature of *Lr42*-mediated resistance to *Pt.*-THSN, we performed silencing of *Pt31812* by BSMV-HIGS approach in *Lr42*-harboring wheat line followed by inoculation with *Pt.*-THSN isolate (Figure 6A). The expression of *Pt31812* was significantly reduced at 24, 48, and 120 hpi in the *Lr42*-harboring wheat line upon treatment with BSMV:*Pt31812*, as compared to the BSMV:00 control (Figure 6A). Remarkably, BSMV-HIGS of *Pt31812* altered the infection phenotype of *Pt.*-THSN on *Lr42*-barboring wheat line, resulting in the formation of *Pt*. pustule on leaf surface of the wheat line, as compared to BSMV:00-treated and other control plants (Figure 6B). Silencing of *Pt31812* therefore rendered the loss of *Lr42*-mediated resistance to *Pt.-*THSN, suggesting a possibility that *Pt31812* serves as an avirulence determining factor for *Lr42*-mediated resistance in wheat.

**FIGURE 7:**
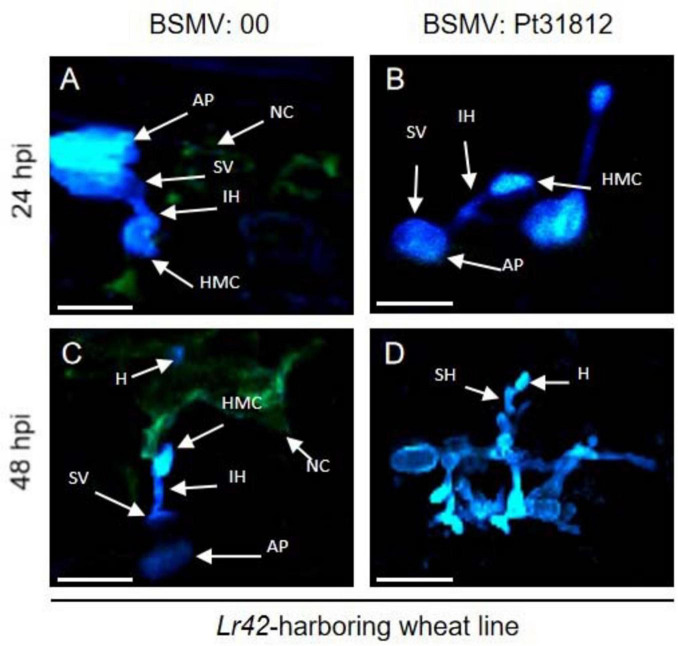
Silencing of *Pt31812* results in loss of *Lr42*-mediated cell death in wheat. (A–D): Confocal imaging of *Pt.*-THSN infected leaf cells of *Lr42*-harboring wheat line at 24 hpi and 48 hpi. Wheat leaves were treated with BSMV empty vector (BSMV:00) or silencing vector (BSMV: Pt31812) followed by inoculation of *Pt*.-THSN uredospores. AP: appresorium; IH: infection hypha; SV: substomatal vesicle; HMC: haustorial mother cell; SH: second hypha; H: haustorium; NC: necrotic cell. Scale bar = 30 μm.

We further followed the growth and development of the *Pt.* fungus in *Lr42*-harboring wheat leaves treated with BSMV:00 or BSMV:Pt31812. After inoculated with uredospores of *Pt*.-THSN isolate, formation of rust appressorium, substomatal vesicle, infection hypha, and haustorial mother cell were observed already at 24 hpi in leaves of both BSMV:00 and BSMV:Pt31812 treated plants (Figures 7A,B), however, necrosis was only observed around the haustorial mother cells in BSMV:00 but not BSMV:Pt31812 treated wheat leaves (Figures 7A,B). Later at 48 hpi, necrosis was persistent and expanded in BSMV:00 treat wheat leaves, along with fewer fungal structures in infected area (Figure 7C). By contrast, necrosis was rarely observed in BSMV:Pt31812 treated wheat leaves at 48 hpi, but formation of fungal structures was significantly increased with development of more fungal mycelia (Figure 7D). These data demonstrated that *Lr42*-harboring wheat line confer resistance to *Pt*.-THSN isolate, and silencing of *Pt31812* rendered loss of *Lr42-*mediated resistance thus converted an *Lr42-*specific avirulent isolate to a virulent isolate.

Taken together, these results suggest that Pt31812 play an avirulence determinant role during *Pt*.-THSN infection of *Lr42*-harboring wheat line.

## 4 Discussion

### 4.1 Pt31812 may be an intracellular effector protein

Pathogen effectors can be broadly categorized into apoplastic effectors functioning in the extracellular space and cytoplasmic effectors delivered into host cells. Among these, intracellular effectors have been more extensively documented for their functions (Dou et al., 2008). For example, the avirulence effectors *AvrL567* and *AvrM* of *M. lini* can be transferred into host cells to exert functions independent of pathogen presence (Rafiqi et al., 2010). Previous studies showed that effectors are localized in plant cell membrane (Lewis et al., 2014), cytoplasm (Zhang et al., 2015) and nucleus (Escoll et al., 2016). In this study, Agrobacterium-mediated transient expression in *N. benthamian* also revealed that Pt31812 is located to cytoplasm and nucleus in plant cell. Thus, we conclude that Pt31812 is an intracellular effector protein that is transported into the cell via a complex mechanism and functions within the host cell.

The transport mechanism of intracellular effector proteins has been the focal point of effector protein research. The RXLR motif is known to be responsible for transporting effectors into host cells in *Oomycetes* (Whisson et al., 2007), while [Y/F/W]xC motif of *Powdery Mildew* is characterized as a putative motif required for host intracellular localization (Godfrey et al., 2010). In this study, no RXLR motif was identified in Pt31812, wheras a [Y/F/W]xC motif was detected at its N-terminus. Notably, similar [Y/F/W]xC-containing effectors in *Pst* have been reported, and point mutation analysis has revealed that the [Y/F/W]xC motif cannot be responsible for translocation of effectors (Cheng, 2015). In this study, Pt31812 contained the YxC motif, and its potential role in the transport of effector proteins in wheat leaf rust requires further investigation.

### 4.2 Pt31812 plays an avirulent role during the infection of *Lr42-*harboring wheat line by THSN

Identifying new avirulence genes and investigating the interaction mechanisms between avirulence and resistance genes are crucial for developing new disease resistance strategies (Gururani et al., 2012). The discovery of *AvrSr35* (Salcedo et al., 2017) and *AvrSr50* (Chen et al., 2017) in *Pgt* provides new research insights. We constructed a recombinant expression vector based on published *Lr1*, *Lr10*, and *Lr21* sequences and co-expressed with *Pt31812 in N. benthemmian*. However, co-expression of *Pt31812* with *Lr1*, *Lr10* or *Lr21* failed to induce cell death, indicating that *Pt31812* is not an avirulent gene for *Lr1*, *Lr10*, or *Lr21*. As most wheat leaf rust resistance genes have not yet been cloned, the co-expression method used for *AvrSr35* (Salcedo et al., 2017) and *AvrSr50* (Chen et al., 2017) cannot currently be applied to explore the recognition between resistance genes and effectors. Therefore, we transiently expressed Pt31812 in 41 near-isogenic wheat lines (single-gene lines) with a Thatcher genetic background to determine whether it could be recognized by the corresponding resistance genes and induce hypersensitive cell death. The results demonstrated that Pt31812 induced HR response in the Lr42 single-gene line. Furthermore, upon infection of Lr42-harboring wheat with *Pt*, silencing of Pt31812 enhanced the virulence of *Pt*. race THSN. Collectively, these results indicating that Pt31812 may be an avirulence gene for *Lr42.*

Previous studies have found that two unrelated Type III effect genes (Avr Rpm1 and Avr B) of bacteria *P. syringae* can also be recognized and interacted by the same Arabidopsis disease-resistant gene (RPM1), while studies have found that the plant protein that directly interacts with Avr Rpm1 and Avr B protein is RIN4 instead of RPM1 (Mackey et al., 2003). Studies indicate that RIN4 is a guardee protein, targeted by Avr Rpm1 and Avr B, and is guarded by RIN4 (Mackey et al., 2003; Axtell and Staskawicz, 2003). This supports the “guard model” for the interaction between avirulence proteins and resistance proteins (Van der Biezen and Jones, 1998). RIN4 is also guarded by another R protein, RPS2, enabling the recognition of other distinct bacterial effector proteins (Mackey et al., 2003). In our study, Pt31812 can induce HR-like cell necrosis in *Lr42*-harboring wheat line, so we speculate that there is a guard protein similar to RIN4 in wheat, which can be targeted by the effector protein Pt31812 and protected by Lr42 disease-resistant protein.

The avirulent function of *Pt31812* was demonstrated when the infection type of the *Lr42*-harboring wheat line inoculated with THSN shifted from low to high after silencing. The leaf rust resistance gene *Lr42* was identified from accession TA2450 in a collection of the wheat wild relative *Aegilops tauschii* Coss. (DD, 2n = 14), the diploid D-genome donor for hexaploid bread wheat (*Triticum aestivum* L., AABBDD, 2n = 42) (Cox et al., 1994). *Lr42* confers all-stage resistance to leaf rust. Since the *Lr42* gene has been cloned, we can verify the avirulent function of the effector protein Pt31812 through co-expression in the future, and then determine whether *Pt31812* is an avirulence gene for *Lr42* or not.

## Data Availability

The original contributions presented in this study are included in this article/Supplementary material, further inquiries can be directed to the corresponding author.

## Footnotes

1
http://www.cbs.dtu.dk/services/SignalP/

2
http://www.cbs.dtu.dk/services/TargetP/

3
http://www.cbs.dtu.dk/services/TMHMM/

4
http://pfam.xfam.org/

5
http://memesuite.org/

## References

[B1] Abebe W. (2021). Wheat leaf rust disease management: A review. *J. Plant Pathol. Microbiol.* 8 1–14. 10.35248/2157-7471.21.12.554

[B2] Anderson C. Khan M. A. Catanzariti A. M. Jack C. A. Nemri A. Lawrence G. J. (2016). Genome analysis and avirulence gene cloning using a high-density RADseq linkage map of the flax rust fungus, *Melampsora lini*. *BMC Genom.* 17:667. 10.1186/s12864-016-3011-9 27550217 PMC4994203

[B3] Axtell M. J. Staskawicz B. J. (2003). Initiation of RPS2-specified disease resistance in *Arabidopsis* is coupled to the AvrRpt2-directed elimination of RIN4. *Cell* 112 369–377. 10.1016/s0092-8674(03)00036-9 12581526

[B4] Catanzariti A. M. Dodds P. N. Lawrence G. J. Ayliffe M. A. Ellis J. G. (2006). Haustorially expressed secreted proteins from flax rust are highly enriched for avirulence elicitors. *Plant Cell* 18 243–256. 10.1105/tpc.105.035980 16326930 PMC1323496

[B5] Chang J. Mapuranga J. Li R. Zhang Y. Shi J. Yan H. (2024). Wheat leaf rust fungus effector protein Pt1641 is avirulent to TcLr1. *Plants (Basel)* 13:2255. 10.3390/plants13162255 39204691 PMC11359021

[B6] Chen J. Upadhyaya N. M. Ortiz D. Sperschneider J. Li F. Bouton C. (2017). Loss of AvrSr50 by somatic exchange in stem rust leads to virulence for Sr50 resistance in wheat. *Science* 358 1607–1610. 10.1126/science.aao4810 29269475

[B7] Cheng Y. Wu K. Yao J. Li S. Wang X. Huang L. (2017). PSTha5a23, a candidate effector from the obligate biotrophic pathogen *Puccinia striiformis* f. sp. tritici, is involved in plant defense suppression and rust pathogenicity. *Environ. Microbiol.* 19 1717–1729. 10.1111/1462-2920.13610 27871149

[B8] Cheng Y. L. (2015). *Identification and functional analysis of pathogenicity-related genes of Puccinia striiformis f.sp. tritici.* Shanxi: Northwest A & F University.

[B9] Cox T. S. Raupp W. J. Gill B. S. (1994). Leaf rust-resistance genes Lr41, Lr42, and Lr43 transferred from *Triticum tauschii* to common wheat. *Crop. Sci.* 34 339–343. 10.2135/cropsci1994.0011183X003400020005x

[B10] Cui Z. Shen S. Meng L. Sun X. Jin Y. Liu Y. (2024). Evasion of wheat resistance gene Lr15 recognition by the leaf rust fungus is attributed to the coincidence of natural mutations and deletion in AvrLr15 gene. *Mol. Plant Pathol.* 25:e13490. 10.1111/mpp.13490 38952297 PMC11217590

[B11] Dagvadorj B. Ozketen A. C. Andac A. Duggan C. Bozkurt T. O. Akkaya M. S. (2017). A *Puccinia striiformis* f. sp. tritici secreted protein activates plant immunity at the cell surface. *Sci. Rep.* 7:1141. 10.1038/s41598-017-01100-z 28442716 PMC5430700

[B12] Dodds P. N. Lawrence G. J. Catanzariti A. M. Teh T. Wang C. I. Ayliffe M. A. (2006). Direct protein interaction underlies gene-for-gene specificity and coevolution of the flax resistance genes and flax rust avirulence genes. *Proc. Natl. Acad. Sci. U. S. A.* 103 8888–8893. 10.1073/pnas.0602577103 16731621 PMC1482673

[B13] Dou D. Kale S. D. Wang X. Chen Y. Wang Q. Wang X. (2008). Conserved C-terminal motifs required for avirulence and suppression of cell death by *Phytophthora sojae* effector Avr1b. *Plant Cell* 20 1118–1133. 10.1105/tpc.107.057067 18390593 PMC2390733

[B14] Escoll P. Mondino S. Rolando M. Buchrieser C. (2016). Targeting of host organelles by pathogenic bacteria: A sophisticated subversion strategy. *Nat. Rev. Microbiol.* 14 5–19. 10.1038/nrmicro.2015.1 26594043

[B15] Geng H. M. Zhang Y. J. Qin Z. Wang S. Liu C. Cui Z. (2024). Puccinia triticina effector Pt-1234 modulates wheat immunity by targeting transcription factor TaNAC069 via its C subdomain. *Crop J.* 13 69–78. 10.1016/j.cj.2024.07.013

[B16] Godfrey D. Böhlenius H. Pedersen C. Zhang Z. Emmersen J. Thordal-Christensen H. (2010). Powdery mildew fungal effector candidates share N-terminal Y/F/WxC-motif. *BMC Genom.* 11:317. 10.1186/1471-2164-11-317 20487537 PMC2886064

[B17] Gu B. Kale S. D. Wang Q. Wang D. Pan Q. Cao H. (2011). Rust secreted protein Ps87 is conserved in diverse fungal pathogens and contains a RXLR-like motif sufficient for translocation into plant cells. *PLoS One* 6:e27217. 10.1371/journal.pone.0027217 22076138 PMC3208592

[B18] Gururani M. A. Venkatesh J. Upadhyaya C. P. Nookaraju A. Kumar Pandey S. Won Park S. (2012). Plant disease resistance genes: Current status and future directions. *Physiol. Mol. Plant Pathol.* 78 51–65. 10.1016/j.pmpp.2012.01.002

[B19] Huerta-Espino J. Singh R. P. Germán S. McCallum B. D. Park R. F. Chen W. Q. (2011). Global status of wheat leaf rust caused by *Puccinia triticina*. *Euphytica* 179 143–160. 10.1007/s10681-011-0361-x

[B20] Kang Z. S. Wang Y. Huang L. L. Wei G. R. Zhao J. (2003). Histology and ultrastructure of incompatible combination between *Puccinia striiformis* and wheat cultivars with low reaction type resistance. *J. Integr. Agr.* 2 1102–1113. CNKI:SUN:ZGNX.0.2003-10-006.

[B21] Kemen E. Kemen A. Ehlers A. Voegele R. Mendgen K. (2013). A novel structural effector from rust fungi is capable of fibril formation. *Plant J.* 75 760–780. 10.1111/tpj.12237 23663217

[B22] Kemen E. Kemen A. C. Rafiqi M. Hempel U. Mendgen K. Hahn M. (2005). Identification of a protein from rust fungi transferred from haustoria into infected plant cells. *Mol. Plant Microbe Interact.* 18 1130–1139. 10.1094/MPMI-18-1130 16353548

[B23] Kolmer J. A. (2005). Tracking wheat rust on a continental scale. *Curr. Opin. Plant Biol.* 8 441–449. 10.1016/j.pbi.2005.05.001 15922652

[B24] Kolmer J. Ordoñez M. Groth J. (2009). “The rust fungi,” in *Encyclopedia of Life Sciences* (ELS). Chichester: John Wiley & Sons Ltd.

[B25] Lacomme C. Santa Cruz S. (1999). Bax-induced cell death in tobacco is similar to the hypersensitive response. *Proc. Natl. Acad. Sci. U. S. A.* 96 7956–7961. 10.1073/pnas.96.14.7956 10393929 PMC22169

[B26] Lewis J. D. Wilton M. Mott G. A. Lu W. Hassan J. A. Guttman D. S. (2014). Immunomodulation by the *Pseudomonas syringae* HopZ type III effector family in *Arabidopsis*. *PLoS One* 9:e116152. 10.1371/journal.pone.0116152 25546415 PMC4278861

[B27] Liu C. Pedersen C. Schultz-Larsen T. Aguilar G. B. Madriz-Ordeñana K. Hovmøller M. S. (2016). The stripe rust fungal effector PEC6 suppresses pattern-triggered immunity in a host species-independent manner and interacts with adenosine kinases. *N. Phytol.* 10.1111/nph.14034 [Epub ahead of print]. 27252028

[B28] Lorrain C. Petre B. Duplessis S. (2018). Show me the way: Rust effector targets in heterologous plant systems. *Curr. Opin. Microbiol.* 46 19–25. 10.1016/j.mib.2018.01.016 29454191

[B29] Lovelace A. H. Dorhmi S. Hulin M. T. Li Y. Mansfield J. W. Ma W. (2023). Effector identification in plant pathogens. *Phytopathology* 113 637–650. 10.1094/PHYTO-09-22-0337-KD 37126080

[B30] Mackey D. Belkhadir Y. Alonso J. M. Ecker J. R. Dangl J. L. (2003). *Arabidopsis* RIN4 is a target of the type III virulence effector AvrRpt2 and modulates RPS2-mediated resistance. *Cell* 112 379–389. 10.1016/s0092-8674(03)00040-0 12581527

[B31] Outram M. A. Chen J. Broderick S. Li Z. Aditya S. Tasneem N. (2024). AvrSr27 is a zinc-bound effector with a modular structure important for immune recognition. *N. Phytol.* 243 314–329. 10.1111/nph.19801 38730532

[B32] Pretsch K. Kemen A. Kemen E. Geiger M. Mendgen K. Voegele R. (2013). The rust transferred proteins-a new family of effector proteins exhibiting protease inhibitor function. *Mol. Plant Pathol.* 14 96–107. 10.1111/j.1364-3703.2012.00832.x 22998218 PMC6638633

[B33] Qi Y. Li J. Mapuranga J. Zhang N. Chang J. Shen Q. (2023). Wheat leaf rust fungus effector Pt13024 is avirulent to TcLr30. *Front. Plant Sci.* 13:1098549. 10.3389/fpls.2022.1098549 36726676 PMC9885084

[B34] Rafiqi M. Bernoux M. Ellis J. G. Dodds P. N. (2009). In the trenches of plant pathogen recognition: Role of NB-LRR proteins. *Semin. Cell Dev. Biol.* 20 1017–1024. 10.1016/j.semcdb.2009.04.010 19398031

[B35] Rafiqi M. Gan P. H. Ravensdale M. Lawrence G. J. Ellis J. G. Jones D. A. (2010). Internalization of flax rust avirulence proteins into flax and tobacco cells can occur in the absence of the pathogen. *Plant Cell* 22 2017–2032. 10.1105/tpc.109.072983 20525849 PMC2910983

[B36] Roelfs A. P. Martens J. W. (1988). An international system of nomenclature for *Puccinia striiformis* f.sp. tritici. *Phytopathology* 78 526–533. 10.1094/Phyto-78-526

[B37] Salcedo A. Rutter W. Wang S. Akhunova A. Bolus S. Chao S. (2017). Variation in the AvrSr35 gene determines Sr35 resistance against wheat stem rust race Ug99. *Science* 358 1604–1606. 10.1126/science.aao7294 29269474 PMC6518949

[B38] Savary S. Willocquet L. Pethybridge S. J. Esker P. McRoberts N. Nelson A. (2019). The global burden of pathogens and pests on major food crops. *Nat. Ecol. Evol.* 3 430–439. 10.1038/s41559-018-0793-y 30718852

[B39] Segovia V. Bruce M. Rupp J. Huang L. Bakkeren G. Trick H. N. (2016). Two small secreted proteins from *Puccinia triticina* induce reduction of ÃŸ-glucoronidase transient expression in wheat isolines containing Lr9, Lr24 and Lr26. *Can. J. Plant Pathol.* 38 91–102. 10.1080/07060661.2016.1150884

[B40] Tang C. L. (2013). *Characterization and functional analyses of effectors and host cell death inducing genes in wheat and Puccinia striiformis interactions.* PhD thesis, Northwest A & F University: Shanxi.

[B41] Upadhyaya N. M. Mago R. Panwar V. Hewitt T. Luo M. Chen J. (2021). Genomics accelerated isolation of a new stem rust avirulence gene-wheat resistance gene pair. *Nat. Plants* 7 1220–1228. 10.1038/s41477-021-00971-5 34294906

[B42] Upadhyaya N. M. Mago R. Staskawicz B. J. Ayliffe M. A. Ellis J. G. Dodds P. N. (2014). A bacterial type III secretion assay for delivery of fungal effector proteins into wheat. *Mol. Plant Microbe Interact.* 27 255–226. 10.1094/MPMI-07-13-0187-FI 24156769

[B43] Van der Biezen E. A. Jones J. D. (1998). Plant disease-resistance proteins and the gene-for-gene concept. *Trends Biochem. Sci.* 23 454–456. 10.1016/s0968-0004(98)01311-5 9868361

[B44] Wang B. Chang J. Mapuranga J. Zhao C. Wu Y. Qi Y. (2024). Effector Pt9226 from *Puccinia triticina* presents a virulence role in wheat line TcLr15. *Microorganisms* 12:1723. 10.3390/microorganisms12081723 39203565 PMC11357290

[B45] Wang F. Shen S. S. Cui Z. C. Yuan S. Qu C. Jia H. (2023). *Puccinia triticina* effector protein Pt_21 interacts with wheat thaumatin-like protein TaTLP1 to inhibit its antifungal activity and suppress wheat apoplast immunity. *Crop J.* 11 1431–1440. 10.1016/j.cj.2023.04.006

[B46] Wang X. Wang X. Deng L. Chang H. Dubcovsky J. Feng H. (2014). Wheat TaNPSN SNARE homologues are involved in vesicle-mediated resistance to stripe rust (*Puccinia striiformis* f. sp. tritici). *J. Exp. Bot.* 65 4807–4820. 10.1093/jxb/eru241 24963004 PMC4144766

[B47] Wang X. Yang B. Li K. Kang Z. Cantu D. Dubcovsky J. (2016). A conserved *Puccinia striiformis* protein interacts with wheat NPR1 and reduces induction of pathogenesis-related genes in response to pathogens. *Mol. Plant Microbe Interact.* 29 977–989. 10.1094/MPMI-10-16-0207-R 27898286

[B48] Whisson S. C. Boevink P. C. Moleleki L. Avrova A. O. Morales J. G. Gilroy E. M. (2007). A translocation signal for delivery of oomycete effector proteins into host plant cells. *Nature* 450 115–118. 10.1038/nature06203 17914356

[B49] Wu J. Q. Sakthikumar S. Dong C. Zhang P. Cuomo C. A. Park R. F. (2017). Comparative genomics integrated with association analysis identifies candidate effector genes corresponding to Lr20 in phenotype-paired *Puccinia triticina* isolates from Australia. *Front. Plant Sci.* 8:148. 10.3389/fpls.2017.00148 28232843 PMC5298990

[B50] Yuan C. Li C. Yan L. Jackson A. O. Liu Z. Han C. (2011). A high throughput barley stripe mosaic virus vector for virus induced gene silencing in monocots and dicots. *PLoS One* 6:e26468. 10.1371/journal.pone.0026468 22031834 PMC3198768

[B51] Zhang M. Li Q. Liu T. Liu L. Shen D. Zhu Y. (2015). Two cytoplasmic effectors of Phytophthora sojae regulate plant cell death via interactions with plant catalases. *Plant Physiol.* 167 164–175. 10.1104/pp.114.252437 25424308 PMC4281015

[B52] Zhang Y. Wei J. Qi Y. Li J. Amin R. Yang W. (2020). Predicating the effector proteins secreted by *Puccinia triticina* through transcriptomic analysis and multiple prediction approaches. *Front. Microbiol.* 11:538032. 10.3389/fmicb.2020.538032 33072007 PMC7536266

[B53] Zhou J. M. Chai J. (2008). Plant pathogenic bacterial type III effectors subdue host responses. *Curr. Opin. Microbiol.* 11 179–185. 10.1016/j.mib.2008.02.004 18372208

