# Supplementary material for: Effector Pt31812 from Puccinia triticina acts as avirulence factor for Lr42-mediated resistance in wheat
Source: Front Microbiol. 2025 Jul 18;16:1570072. doi: 10.3389/fmicb.2025.1570072 (PMC12313645; doi:10.3389/fmicb.2025.1570072)
Supplement: Supplementary file 1 [file Data_Sheet_1.docx]

**Supplementary Data S1 Sequence information for Pt31812**

Nucleic acid sequence of Pt31812

ATGAAAGCTACCGTCGTGGGCATCCTGCTGGCCGCTTTTGCGCTTGCCTGCGTGAAGGCAGACAACGAGCACACCCAATGCTACAAATACTTCTTGAACAAAGACCATTGTGTCCAGGCTACCGGCGTTGAGGCCGACAGATGTACTAAGAAGGAGTCGCACGAACTGGAATCAGTCCGACTGATGACGATACAGTCTGGAGGTAGCGGGCGCTTGCAGCGTCGTTACAACGGGCCGGCCACGGTTGCCGAGCACGCTACGCAGATCTACTCATGTCCCGGCCTGAAGCCCGACCCTAATGGGTATGAAGTGTGTGTCTGGGCTGGGGATGGAAATGGGTCCAAGAACACTGGTTGGCTCGACAAGGCCAACAAGGTCAACTGCGGGAAGCAAGTTTACATCCAACGAAAAGATGACAAAAAGAGTGTCCAATATGCCACGATTACCGGTGGCTGTGATTTCGGGAATGTGAAGCCCGAGTCGGGATGTTTCCAGATCGGGATCAACCAGCCGCTCTTCGAGTCATTCAAGCCCTCGCAGGGCGAAAAGGACAAAAAACTAATCGCTTCAATGACCTGGGACTTCAACAACCTCAACTTCGACAACCCAAAGAACGCAGCCTACTGA

Protein sequence of Pt31812

MKATVVGILLAAFALACVKADNEHTQCYKYFLNKDHCVQATGVEADRCTKKESHELESVRLMTIQSGGSGRLQRRYNGPATVAEHATQIYSCPGLKPDPNGYEVCVWAGDGNGSKNTGWLDKANKVNCGKQVYIQRKDDKKSVQYATITGGCDFGNVKPESGCFQIGINQPLFESFKPSQGEKDKKLIASMTWDFNNLNFDNPKNAAY

Supplementary Table S1 Primers designed for this study

| Primer name | Primer sequence |
| --- | --- |
| ORF31812-F | 5′-ATGAAAGCTACCGTCGTGGG-3′ |
| ORF31812-R | 5′-TCAGTAGGCTGCGTTCTTTG-3′ |
| U31812-F | 5'-TCCCCCGGGATGAAAGCTACCGTCGTGGG-3' |
| △SP31812-F | 5'-CCCATCGATGACAACGAGCACACCCAATG-3' |
| U31812-R | 5'-GCGTCGACTCAGTAGGCTGCGTTCTTTG-3' |
| RTU31812-F | 5′-ACAAGGCCAACAAGGTCAAC-3′ |
| RTU31812-R | 5′-TTCACATTCCCGAAATCACA-3′ |
| EF1a-F | 5'-TCGTGTCGAAACCGGTACCATCAA -3' |
| EF1a-R | 5'-AAACCAACGTTGTCACCTGGCAT-3' |
| DW31812-F | 5′- CCCATCGATGACAACGAGCACACCCAATG-3′ |
| DW31812-R | 5′- TCCCCCGGGGTAGGCTGCGTTCTTTGGGT -3′ |
| V31812-F | 5′- AAGGAAGTTTAAATCCATCCACCTTCTCCAGC-3′ |
| V31812-R | 5′- AACCACCACCACCGTGATTCCAGTTCGTGCGACTC-3′ |


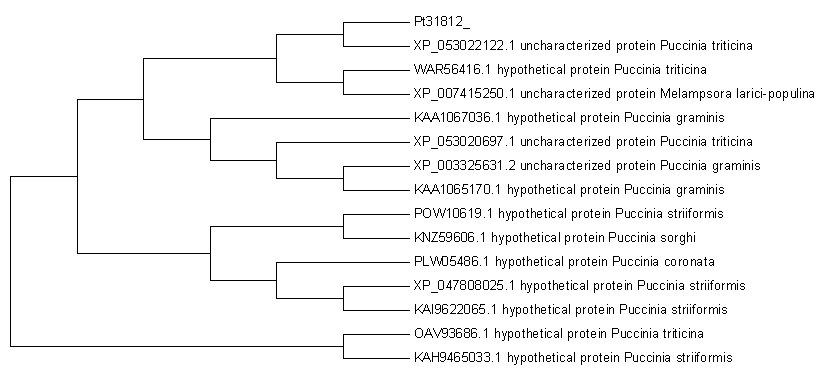


A

B


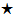


**Supplementary Figure S1. Sequence alignment and phylogenetic tree of Pt31812**

1. In NCBI, 15 homologous proteins were screened by blastP, and the sequences were compared by DNAMAN. The results of sequence alignment showed that [Y/F/W]xC motifs were found in other homologous sequences of *Puccinia triticina*, *Puccinia graminis* and *Puccinia striiformis*.
2. The phylogenetic tree of pt31812 was constructed by MEGA. The results showed that pt31812 and IXP_053022122.1 uncharacterized protein Puccinia triticina had the closest genetic relationship.


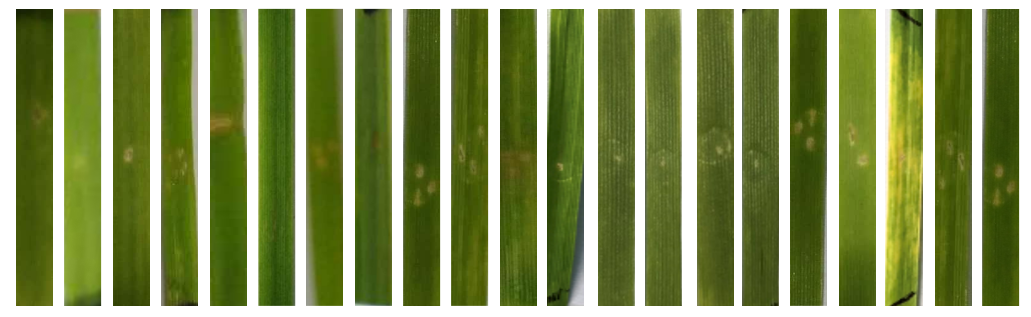

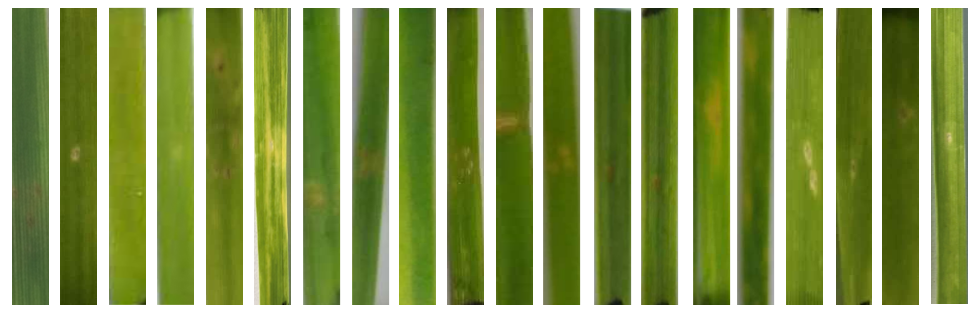


1 2 3 4 5 6 7 8 9 10 11 12 13 14 15 16 17 18 19 20 21

22 23 24 25 26 27 28 29 30 31 32 33 34 35 36 37 38 39 40 41 21

**Supplementary Figure S2. Pt31812 induced cell death in the Lr42 and TcLr25.**

The effector proteins were delivered into the 41 wheat leaf rust resistant near-isogenic lines (monogenic lines). 1-41 of wheat materials was respectively represent TcLr1, TcLr2a, TcLr2c, TcLr3, TcLr9, TcLr16, TcLr24, TcLr26, TcLr3ka, TcLr11, TcLr17, TcLr30, TcLrB, TcLr10, TcLr14a, TcLr18, TcLr21, TcLr28, Lr42, TcLr2b, TcLr3bg, TcLr14b, TcLr15, TcLr19, TcLr20, TcLr23, TcLr25, TcLr29, TcLr27+31, TcLr32, TcLr33, TcLr33+34, TcLr36, TcLr38, TcLr41, TcLr44, TcLr45, TcLr47, TcLr50, TcLr51, and TcLr53.
